# Effects of ozone exposure on human epithelial adenocarcinoma and normal fibroblasts cells

**DOI:** 10.1371/journal.pone.0184519

**Published:** 2017-09-08

**Authors:** Anna Poma, Sabrina Colafarina, Eleonora Aruffo, Osvaldo Zarivi, Antonella Bonfigli, Sebastiano Di Bucchianico, Piero Di Carlo

**Affiliations:** 1 Department of Life, Health and Environmental Sciences, University of L'Aquila, L'Aquila, Italy; 2 Department of Psychological, Health and Territorial Sciences, University "G. d'Annunzio" of Chieti-Pescara, Chieti, Italy; 3 Centre of Excellence CETEMPS, University of L'Aquila, L'Aquila, Italy; Consiglio Nazionale delle Ricerche, ITALY

## Abstract

Previous studies show variable ozone cytotoxicity and genotoxicity in cell cultures, laboratory animals and humans directly exposed to tropospheric ozone. The aim of this study was therefore to investigate and compare the cyto and genotoxic effects of ozone using adenocarcinoma human alveolar basal epithelial cells A549 and normal human fibroblasts Hs27. A cell culture chamber with controlled atmosphere (a simulation reactor) was built to inject a flow of 120 ppb of ozone, which is two times the threshold value for the protection of human health, fixed by the EU legislation. Cell proliferation was evaluated by a luminescent cell viability assay while we assessed the genotoxic potential of ozone by the induction of micronuclei as well as evaluating DNA strand breaks by the induction of micronuclei evaluated by means of the cytokinesis-block micronucleus (CBMN) assay as well as evaluating DNA strand breaks by Alkaline Comet Assay (CA) or Comet Assay. A549 cells viability decreases significantly at 24 hours treatment with 120 ppb of O_3_ while at 48 hours and 72 hours O_3_ treated cells viability doesn’t differ in respect to the control. However a significative decrease of A549 viability is shown at 72 hours vs. 48 hours in both treated and not-treated cells. The viability trend in the Hs27 cells did not show any significant changes in treated samples compared to the control in all conditions. The two genotoxicity biomarkers, the micronucleus and the comet tests, showed in both the cell types exposed to ozone, a significant increase in the number of micronuclei and in the tail DNA % in respect to the control even if at different times/cell type. Moreover, we found that O_3_ provokes genotoxic effects more evident in A549 cancer cells than in normal fibroblasts Hs27 ones. We applied a cell growth simulation model referred to ozone treated or not cell lines to confirm that the ozone exposure causes a slackening in the cells replication.

## Introduction

Surface ozone (O_3_), is an allotropic form of oxygen and it is a well recognize pollutant and greenhouse gas with relevant effects on human health and ecosystems. For instance, the European Union has estimated 17,000 premature deaths each year related to O_3_ concentrations exceeding the annual average of 70 *μ*g/m^3^ and more than 14,000 hospital admissions each year linked to high concentrations of O_3_ [1]. O_3_ is mainly present in the stratosphere, where it has a fundamental importance for the development and maintenance of life on Earth, because it plays a shield function for ultraviolet radiation that has feared health and genetic effects. On the contrary, the percentage of O_3_ present in the troposphere, albeit extremely lower, it is the main responsible of photochemical pollution and, therefore, the consequent "photochemical smog". The tropospheric O_3_ is a typical secondary pollutant, the formation of which is a post-reactions between precursor compounds such as nitrogen oxides (NOx) and volatile organic compounds (VOCs), the latter can be of natural or anthropogenic origin. For example, episodes may occur by intrusion stratospheric O_3_ in the troposphere, in conjunction with particular meteorological events; or, where they occur spontaneous fires, volcanic eruptions or bacterial fermentation processes [2]. Biogenic VOCs are primarily released in atmosphere by biogeochemical processes, enteric fermentation in animals, intensive farming and domestic waste and vegetation. Anthropogenic VOCs sources, are mainly related to industrialized and densely populated areas, where the greatest contribution is given by vehicular traffic, industrial processes and domestic heating. Episodes of photochemical smog are usually associated with high-pressure, typical of the summer months [3,4].

Case-control studies in humans indicate that the levels of O_3_ that can be found in many areas of the world induce functional and biochemical alterations, mostly of the respiratory tract. Although gradual exposure to ozone causes different levels of adaptation, it is plausible that multiple serious injuries may cause permanent damage to the organs at risk. Recent epidemiological studies have confirmed that ozone is associated with acute and negative health effects, both in terms of morbidity and mortality; chronic exposure to ozone to which millions of people are regularly exposed results in significant damages in the airways within the bronchioles. O_3_ is a strong oxidant and in aqueous solutions it decomposes producing reactive oxygen species (ROS) like superoxide, hydroxy radicals and hydrogen peroxide which could change the oxidant/antioxidant balance in the lungs airway lining fluid leading to oxidative stress. Although the oxidative stress due to formation of ROS and ozonation products resulting by the ozone exposure are known the mechanisms by which the oxidative stress is induced by ozone are not yet well established [5,6]. Ozone-induced oxidative stress leads to several events including influx of granulocytic inflammatory cells, activation of alveolar macrophages, damages to airway epithelial cells and to lung tissues and function [7]. Some of the biological processes involved in ozone-induced oxidative stress and injury with respect to reduction of inflammation and beginning of tissue repair were evidenced [8]. The examination of gene expression levels of bronchoalveolar lavage cells after exposure to high levels of ozone, demonstrated that a highly differentially expressed gene (SPP1, the gene for osteopontin) played a role in airway epithelium wound repair after O_3_ exposure induced inflammatory pathways. Previously it has been shown that acute ozone exposition (2 and 5 ppm for 2 hr) induces differential gene expression profiles in rat lung tissue by using a rat cDNA expression array containing 588 characterized genes. Gene array analysis indicated differential expression in most of the genes with roles in the initial toxicity response but also the induction of nine genes specific to 2-ppm (thyroid hormone-*β* receptor c-erb-A-*β* and glutathione reductase) or 5-ppm exposure groups (*c-jun*, induced nitric oxide synthase, macrophage inflammatory protein-2, and heat shock protein 27) [9]. O_3_ induces cardiovascular diseases [10,11], an increase in vascular markers of inflammation and changes in fibrinolytic markers that could potentially induce changes in autonomic control of heart rate [12].

Ozone is genotoxic to cell cultures but past studies of cytogenotoxicity with laboratory test animals after inhalation exposure were contradictory because for example lung adenomas were induced in strain A/J mice but not in Swiss-Webster mice [13] whereas increased levels of genomic instability related to O_3_ exposure of human lymphocytes *in vivo* and *ex vivo* have been reported [14–16]. The available literature on *in vitro* studies dealing with cytotoxicity and genotoxicity of O_3_ is still quite poor; recently [17] the cellular response to ozone exposure has been reported to investigate the *in vitro* effects of low O_3_ concentrations, currently administered in clinical practice (10 and 16 μg O_3_/ml O_2_), on cultured SH-SY5Y neuronal cells. Mild ozonisation did not affect cell viability while molecular analyses showed an O_3_-induced modulation of some genes involved in the cell response to stress (HMOX1, ERCC4, CDKN1A). In [18] conjunctival epithelial cells, used as an in vitro substitute for a mouse model, were exposed, without or with interleukin (IL)-1α (10 ng/mL) pretreatment, to 0.5 ppm and 2.0 ppm of ozone for 1 and 2 hours in an exposure chamber. After 1 and 2 hours of incubation in condition of 0.5 or 2.0 ppm of O_3_ exposure, the cells showed no changes in viability; anyway, the ozone increased the inflammatory response and altered oxidative status and mitochondrial function in IL-1α-pretreated conjunctival epithelial cells. In [19] EpiAirwayTM 3-D cells (human-derived cell cultures of differentiated airway epithelial cells) in comparison to the responses of A549 human alveolar epithelial cells were exposed to 400 ppb of ozone (O_3_) for 4 h. Cytotoxicity, but not genotoxicity, was assessed by measuring lactate dehydrogenase (LDH) release into the culture medium and apical surface. Interleukin 6 (IL-6) and interleukin 8 (IL-8) proteins were measured in the culture medium and in the apical washes to determine the inflammatory response after exposure. O_3_ significantly increased basolateral levels of LDH and IL-8 in A549 cells while no significant changes in LDH and IL-8 levels were observed in the EpiAirwayTM cells. The EpiAirwayTM cells show minimal adverse effects after exposure suggesting that they are more toxicologically resistant compared to A549 cells.

Several mathematical and numerical models have been developed to support and to understand the results of *in vivo* and *in vitro* experiments. Most model have been proposed to describe the population growth [20–26]. In details, Hallam et al. [27] suggested a three-dimensional model describing cell population subjected to toxicants exposures: they described the system with a first order kinetics for the toxicant uptake, a logistic equation of the population growth and a linear dose-response function. Anton et al. [26], evoking the model proposed by Hallam et al.[27], developed a three-dimensional model of differential equations based on the logistic equation in which they considered a loss term dependent on the toxicant concentration; they evaluated the parameters by an Expectation Maximization algorithm and determined the lowest concentration of the toxicant causing cell death. In our study, we adopted and numerically solved the classic logistic model to simulate both the control and the treated A549 cells viability modifying the equation of the logistic growth model (LGM) with the assumption that the growth rate and the carrying capacity are time-dependent.

The aim of this study was the investigation of the cyto and genotoxic effects due to O_3_ exposure using adenocarcinoma human alveolar basal epithelial cells A549 and normal human fibroblasts Hs27. Cell proliferation was evaluated by a luminescent cell viability assay based on ATP quantification as a measure of metabolically active cells. We assessed the genotoxic potential of O_3_ by the induction of micronuclei evaluated by means of the cytokinesis-block micronucleus (CBMN) assay as well as evaluating DNA strand breaks by Alkaline Comet Assay (CA). A cell culture chamber with controlled atmosphere was built to inject a flow of 120 ppb of O_3_ comparable to high pollution days in the urban environment [15].

The results of these experimental studies were then used to model the cells population trend using the classic and well-known logistic growth model [20] to confirm that the ozone exposure causes a slackening in the cell replication.

## Materials and methods

### Ozone source and culture chamber with controlled atmosphere

**Fig 1:**
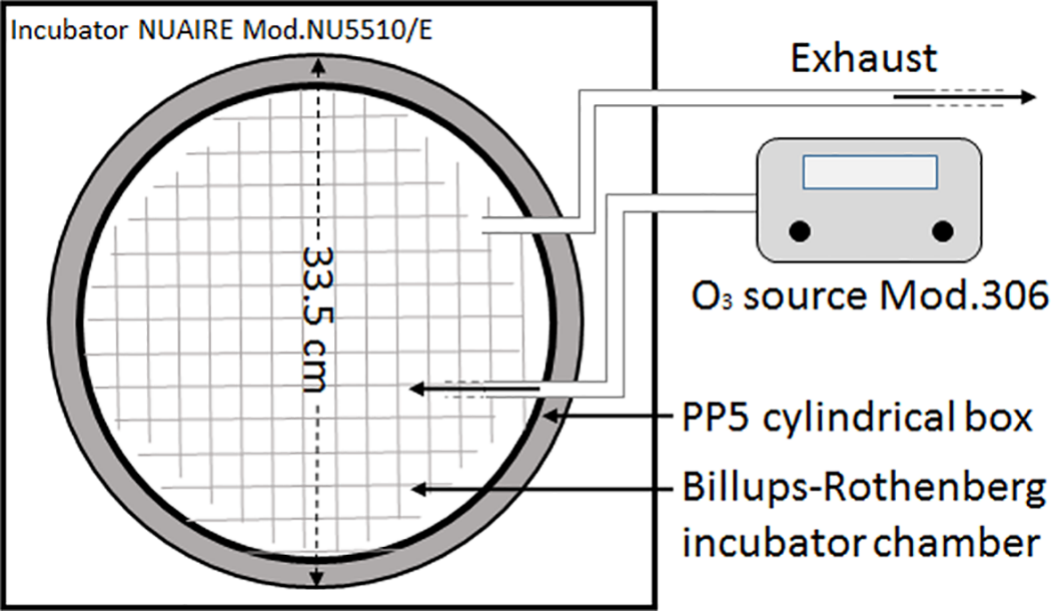
Schematic illustration of the exposure system. The main parts of the exposure system are: an Ozone Calibration Source, **a** PP5 box culture chamber and a modular incubator chamber. The culture and incubator chambers are put in place inside the NUAIRE incubator (Mod. NU5510/E).

The Ozone Calibration Source Model 306 (2B Technologies) was used as an O_3_ and zero air source. The device produces either zero air (oxygen and nitrogen in the air mixing ratio) or 120 ppb of O_3_. The total flow output was 3.0 L/min. A mercury lamp at low pressure was used in the O_3_ calibration source to photolyze oxygen and produce ozone. A cylindrical PP5 (polypropylene) box (∅ 33.5 cm, h 13 cm), with removable lid, has been used as culture chamber. Inside the box we placed the base of a modular incubator chamber (Billups-Rothenberg, box 977 del mar California 92014) with a grid on the bottom to keep the cells at 80% humidity with a water tank. During the experiment the O_3_ was flowed through perfluoroalkoxy (PFA) tube into the cylindrical box and following into the incubator chamber, which have been kept closed with lids in order to guarantee an homogenous concentration of O_3_ during the exposure. An exhaust tube has been used to avoid accumulation of the O_3_ in the chamber and the incubator (Fig 1).

### Cell lines and cell cultures

The human alveolar adenocarcinoma epithelial cells (A549, ATCC CCL-185) and human skin fibroblasts (Hs27, ATCC 1634-CRL) were purchased from American Tissue Type Collection. They have been grown in Dulbecco’s modified Eagle’s medium supplemented with 10% fetal bovine serum, 2 mM L-glutamine, penicillin (100UI/ml) and streptomycin (100 *μ*g/ml), and maintained at 37°C in a humidified atmosphere (95%) under 5% CO_2._ Cells were seeded at approximately 10,000 cells/cm^2^ and passaged every 3–4 days as necessary. Cells were removed from the plates with 0.05% trypsin-0.02% EDTA solution and reseeded 24 h prior the exposures to 120 ppb of O_3_. All cells materials were purchased from Sigma-Aldrich.

### Cell viability assay

CellTiter-Glo® Luminescent Cell Viability Assay (Promega) was used to determine the number of viable cells in culture based on quantification of the ATP according to manufacturer´s instruction. The effects of 120 ppb O_3_ on cell proliferation were evaluated following exposures for 0, 24, 48 and 72 hrs and compared to untreated cells. Adequate positive controls (cells treated with Triton-X-100 0.1%) were run with each set of experiments (24, 48 and 72 hrs).

### Cytokinesis-block micronucleus (CBMN) assay

CBMN was carried out with slight modifications according to the protocol of Fenech et al.[28] and OECD guideline [29]. A total of 2.5 x 10^5^ cells were seeded in each flask and after 24 h of culture, the cells were used for controls and treatments. The cells were exposed to 120 ppb O_3_ for 48 and 72 hrs and colchicine was used as positive control at 5 *μ*g/ml at the same treatment times. Cytochalasin B was added to cell cultures at a final concentration of 3 *μ*g/ml. Cells were harvested 24 h later, washed in PBS, resuspended (about 5 x 10^6^ cells/ml) and spread onto glass slides (20 *μ*l of cell suspension per slide); after air-drying, cells were fixed with methanol/glacial acetic acid (3:1) for 10 min, and stained with 5% Giemsa solution for 5 min. All procedure were conducted at room temperature. After washing with distilled water, the slides were rapidly dried in xylene and mounted with Canadian balsam. By using Leitz light microscope at 400x and 1000x magnification and following the criteria of the OECD guideline [26], 1000 binucleated cells were analysed for each condition. Three biological replicates for each sample were used for CBMN analysis and three technical replicates (slides) were analyzed for each biological replicate. Hs27 and A459 cells were treated for 48 and 72 h with ozone to evaluate its effects on MN induction. Replication Index (RI) was calculated according to [26] as follows:

$$RI=\frac{(\left(No.\;binucleate\;cells)+(2\times No.\;multinucleate\;cells\right))÷{\left(Total\;number\;of\;cells\right)}_{Treated}}{(\left(No.\;binucleate\;cells)+(2\times No.\;multinucleate\;cells\right))÷{\left(Total\;number\;of\;cells\right)}_{Control}}\times 100$$

### Alkaline Comet Assay

Hs27 and A549 cells were treated as described in Cell lines and Cell Cultures sub-section for 48 and 72 h with 120 ppb ozone to evaluate its genotoxic effect on comet formation.

The hydrogen peroxide was used as positive control at 100 μM incubated at 37°C for 1 h. At the end of the incubation the cells were harvested and used in comet assay analysis of DNA damage.

The CA was performed according to the guidelines reported in [30,31]. Briefly, after their detachment with 0.05% trypsin-0.02% EDTA, the cells were washed with PBS, suspended in 0.7% low melting point agarose (LMA) and maintained at 37%. A final concentration of 90,000 cells/ml was obtained and 150 *μ*l of such suspension were spread on a normal agarose pre-coated standard microscope slide and placed at 4° C for 5 min. After agarose solidification, another layer of LMA was added. Slides were immersed in ice-cold freshly prepared solution (2.5 M NaCl, 100 mM Na_2_EDTA, 10 mM Tris, 10% DMSO, 1% Triton X-100, pH 10) to lyse cells and allow DNA unfolding. After 1 h at 4° C in dark condition, slides were placed on a horizontal electrophoresis unit which was filled with fresh buffer (1 mM Na_2_EDTA, 300 mM NaOH, pH 13) to cover the slides. The cells were immersed in the buffer alkaline solution for 20 min to allow DNA unwinding and expression of single-strand breaks and alkali-labile sites. Electrophoresis was performed in the same buffer at 4°C for 20 min by applying an electric current of 300 mA at a voltage of 1 V/cm. All of these steps were conducted under yellow light to prevent the occurrence of additional DNA damage. After electrophoresis the slides were placed horizontally and Tris buffer (0.4 M Tris, pH 7.5) was gently added to neutralize the excess alkali. The slides were allowed to sit for 5 min; this operation repeated three times. Subsequently the slides were air dried and then stored at 4°C until the analysis of the DNA migration. Just before the DNA migration analysis, 100 *μ*l of ethidium bromide (20 *μ*g/ml) was added to each slide that was immediately covered with a coverslip. After 20 min the coverslips were removed and the slides rinsed in distilled water to remove the excess of ethidium bromide. The slides were again covered with coverslips. Observations were conducted at 200x magnification using a Zeiss Axioplan 2 fluorescence microscope equipped with excitation and barrier filters.

Three biological replicates for each sample were used for comet assay and three technical replicates (slides) were prepared from each sample and randomly scanned: 300 cells per slide were analyzed and evaluated for DNA migration. The analysis of comets was performed by using the software CASPLab, the image analysis calculates different parameters for each comet. To evaluate the extent of DNA damage in individual cells, the percentage of DNA in the tail of the comet and Olive Tail Moment were used [30–32]. The Tail Moment represents the percentage of tail DNA multiplied by the tail length divided by100.

### A numerical model of population growth

A classic logistic growth model, introduced by [20], has been selected to numerically model the control cells viability curve for the adenocarcinoma cell line A549. It describes the cells growth by the following ordinary differential equation (ODE):

$$\frac{dN}{dt}\;=\;\alpha \;\left(1-\frac{N}{k}\right)\;N$$ (1)

where N is the population number, *α* is the growth rate and *k* represents the carrying capacity of the cells growth environment. We applied the model in two different scenarios: 1) as first approach, we solved the ODE (1) considering *α* and *k* independent by the time; 2) in second scenario, we introduced a dependence by time of both the growth rate and the carrying capacity supposing that they change during the experiment; in details, we assumed that *α* increases exponentially (*α*(*t*) = *α*_0_*e*^*βt*^) and k decreases (*k*(*t*) = *k*_0_*e*^−*γt*^), which means that, as the population grows, the capacity of the growth environment decreases. Several investigation have been done on the impact of the addition of time-varying parameters in the LGM studying the mathematical properties and, sometimes, giving approximate solutions of the equation [33,34]. In our analysis, we solved numerically the LGM equation with time-dependent parameters. The parameters *α* and *k* (that is, *α*_0_, *k*_0_, *β* and *γ* in the second scenario) have been derived applying the least squares data fitting method to the measured data of the negative control. The treated population, on the contrary, has been modelled solving Eq (1) using the parameters evaluated for the control and modified according to RI (Replication Index) variation (CBMN experiment results) due to the damage produced by the ozone exposure. The model has been initialized knowing the initial population of cells cultured for the cell viability assay test. The parameters evaluation by the least squares method has been constrained in range of values biologically expected.

### Statistical analysis

For each sample, were used three biological replicates run in triplicate. Student's t-test was applied for statistical analysis of viability and micronucleus data to verify whether the average value of the treated conditions differs significantly from a reference value (control). Statistical analyses for the CA, were performed with Mann-Whitney test (non-parametric test): * = p<0,05; ** = p<0,005; *** = p<0,0005 values were considered as statistically significant.

## Results

### Effect of ozone on viability of A549 and Hs27 cells

**Fig 2:**
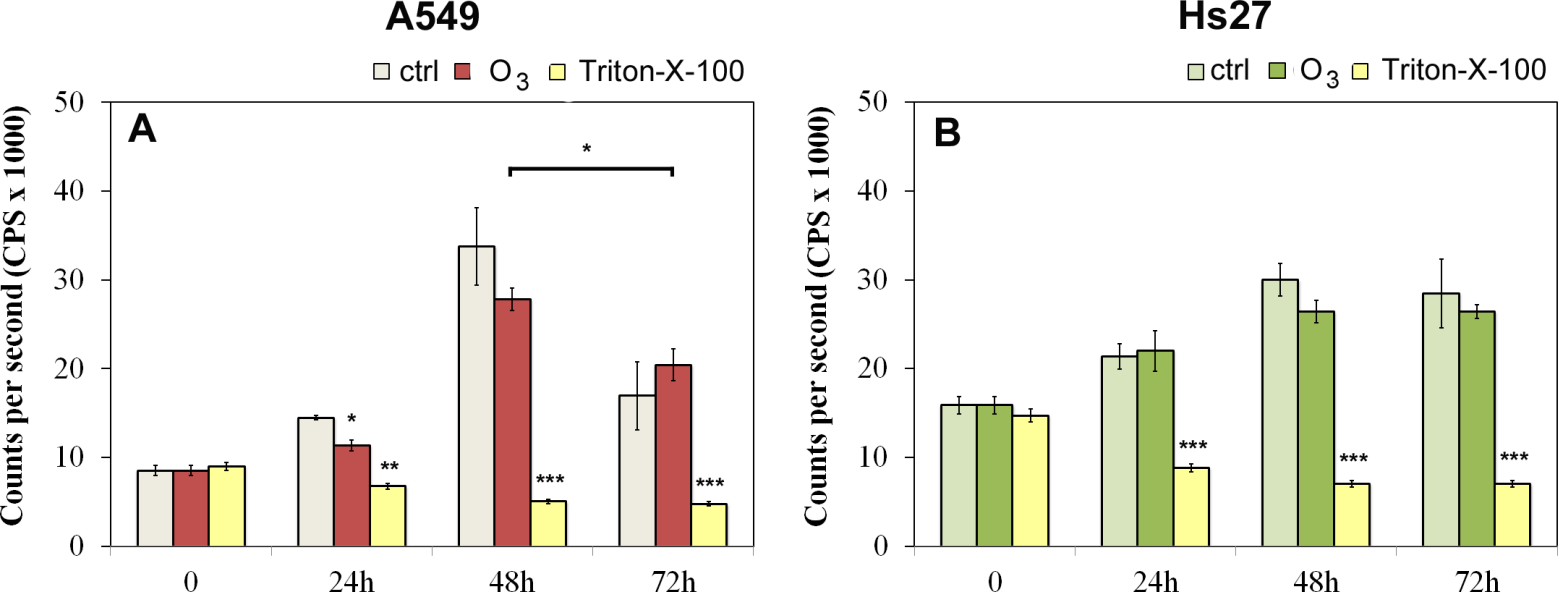
Viability test in A549 and in Hs27 cells. The effects of 120 ppb O_3_ on cell proliferation were evaluated following exposures for 0, 24, 48 and 72 hrs and compared to untreated cells (A549 Fig 2A and Hs27 Fig 2B). Triton-X-100 0.1% was used as positive control. Significance values were determined according to the t-Student: * = p<0,05; ** = p<0,005; *** = p<0,0005 error bars represent the standard error of the mean.

A549 cells viability decreases significantly (21.5%) at 24 hours treatment with 120 ppb of O_3_ treated condition while at 48 hours and 72 hours treated cells viability doesn’t differ in respect to the control (Fig 2A). However a significative decrease of A549 viability is shown at 72 hours vs. 48 hours in both treated (26%) and not-treated cells (50%). Fig 2B shows the viability trend in Hs27 cells: no significant changes in treated samples compared to the control have been revealed from 24 h to 72 hours which indicates that no effect on cell viability was induced by ozonisation.

### Genotoxic effect of ozone on A549 and Hs27 cells investigated by CBMN and Alkaline Comet Assay

**Fig 3:**
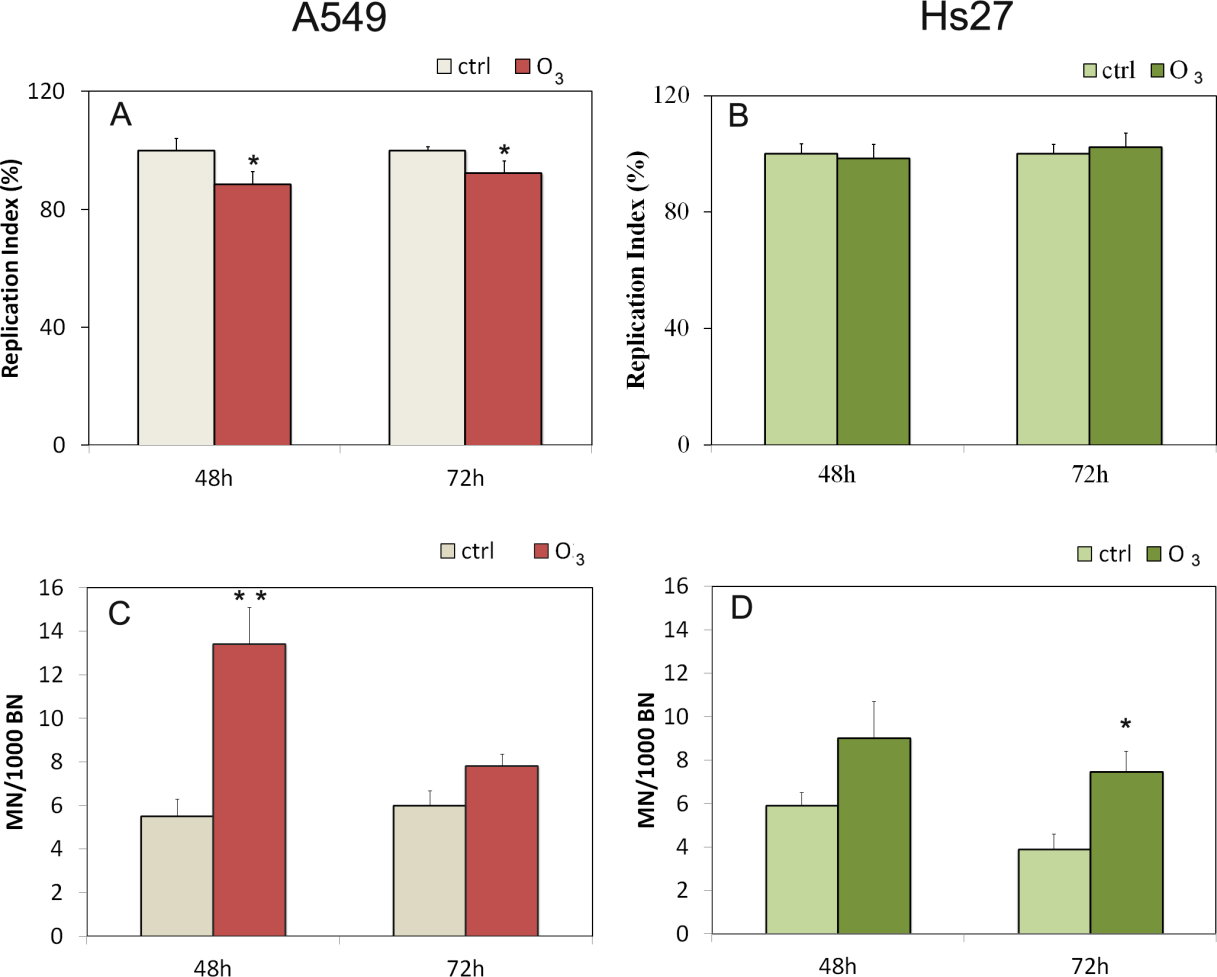
RI Replication Index and micronucleus induction in A549 and Hs27. Induction of cytotoxicity according to RI and genotoxicity according to micronuclei test in A549 cells (panels A and C), and Hs27 cells (panels B and D) in both the condition (control and ozone exposure). Significance values determined according to the t-Student: * = p<0,05; ** = p<0,005; error bars represent the standard error of the mean.

In experiments conducted with cytochalasinB, the cytostasis/cytotoxicity can be quantified from the Replication Index (RI): we used the RI index determination to assess cell proliferation from at least 500 cells per culture (Fig 3 panels A and B). These measurement of cell proliferation have been used to estimate cytotoxicity by comparing values in the treated and control cultures of A549 and Hs27. The RI showed a significant decrease in the presence of 120 ppb O_3_ both at 48 hours (11%) and 72 hours (6%) in A549 cells but not in HS27 so we can assume that 120 ppm ozone was cytotoxic for A549 cultures according to RI values.

The micronucleus test was performed at 48 and 72 hours. In Fig 3C and 3D the numbers of micronuclei referred to 1000 binucleated cells are shown. It is important to observe that in the A549 cells (Fig 3C) at 48 hours of treatment with O_3_, there is a significant increase in the number of micronuclei (about 100% respect to the control) and that this is not found for 72 hours of exposure. On the contrary in the Hs27 treated cells (Fig 3D) significant changes are shown at 72 hours (about 100% respect to the control) but not at 48 hours in the ozone treated samples.

**Fig 4:**
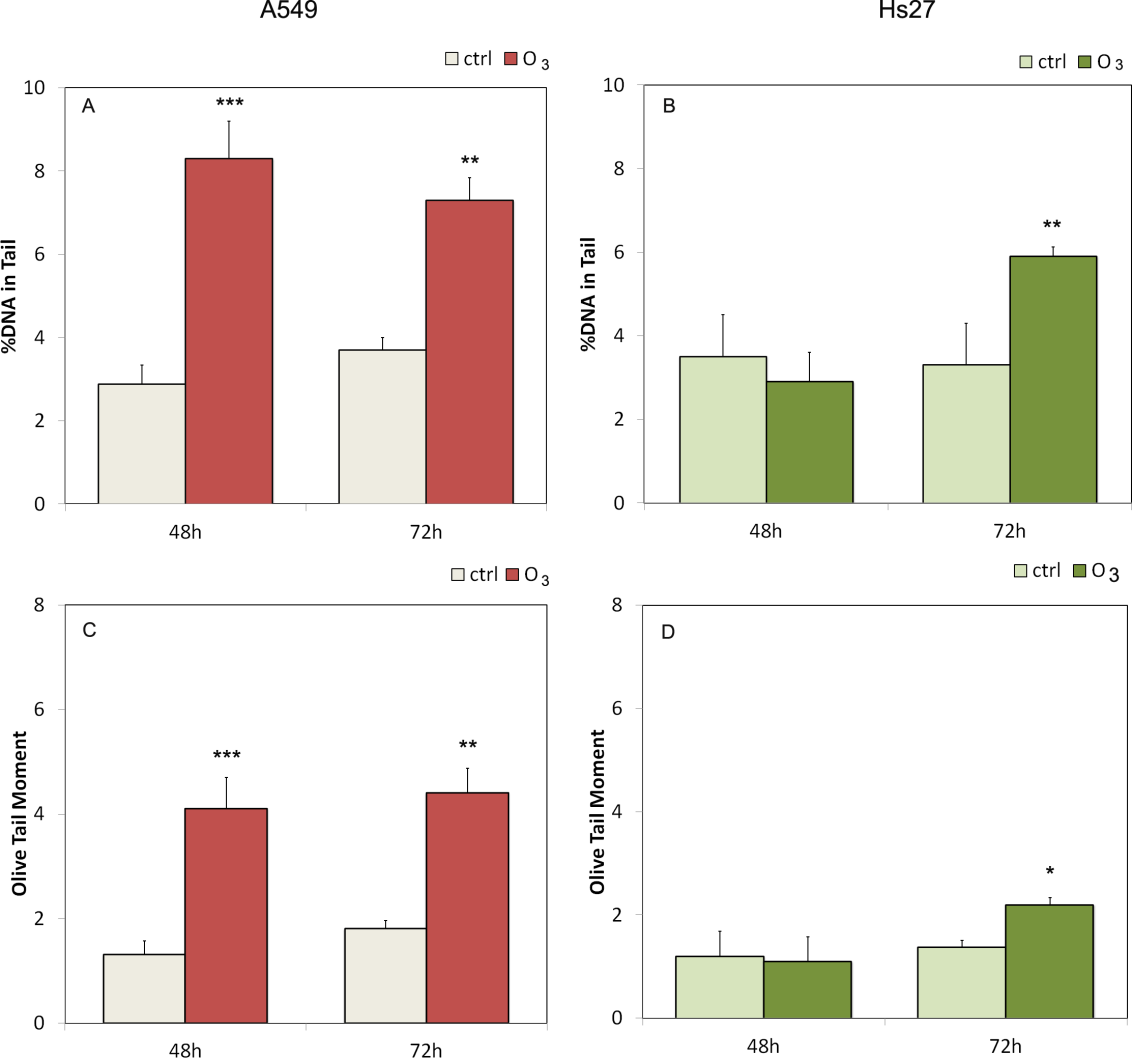
Genotoxic effect of ozone on A549 and Hs27 cells as determined by Comet assay. DNA damage expressed as % DNA in tail (upper panels) and as Olive Tail Moment (lower panels) for the A549 (panels A and C) and the Hs27 (panels B and D) cells. The results obtained for the treated cells with 120 ppb of O_3_ (for 48 and 72 hours) are compared to the negative control; we used the means ± SE calculated for at least three replicates for each experimental point. Statistical significant differences from the untreated controls is given by * (p <0.05), ** (p < 0.005) and *** (p <0.0005). Error bars represent the standard error of the mean.

The Comet Assay was performed at 48 and 72 hours. In Fig 4A /Fig 4B and in Fig 4C/ Fig 4D, the A549 and Hs27 values of tail DNA % and the Olive Tail Moment are shown respectively. We found significant changes in the tail DNA%. In particular A549 cells showed anincrease of the tail DNA % in respect to the control of 8.3% vs 2.88% at 48 hours and 7.3% vs 3.7% at 72 hours. The Olive Tail Moment in A549 cells increased at both ozone exposition times (4.1 ± 0.6 vs 1.3 ± 0.28 at 48h and 4.4± 0.48 vs 1.8±0.16 at 72 h) respect the control (Fig 4C). Hs27 cells (Fig 4B and 4D) at 72 hours only showed increased tail DNA % in respect to the control (5.9% vs 3.3%) and showed increased Olive Tail Moment in respect to control (2.2 ± 0.13 vs 1.38± 0.40). According to the CA the O_3_ is more genotoxic for A549 cells than for Hs27.

### Cell growth simulation models for the A549 cells

**Fig 5:**
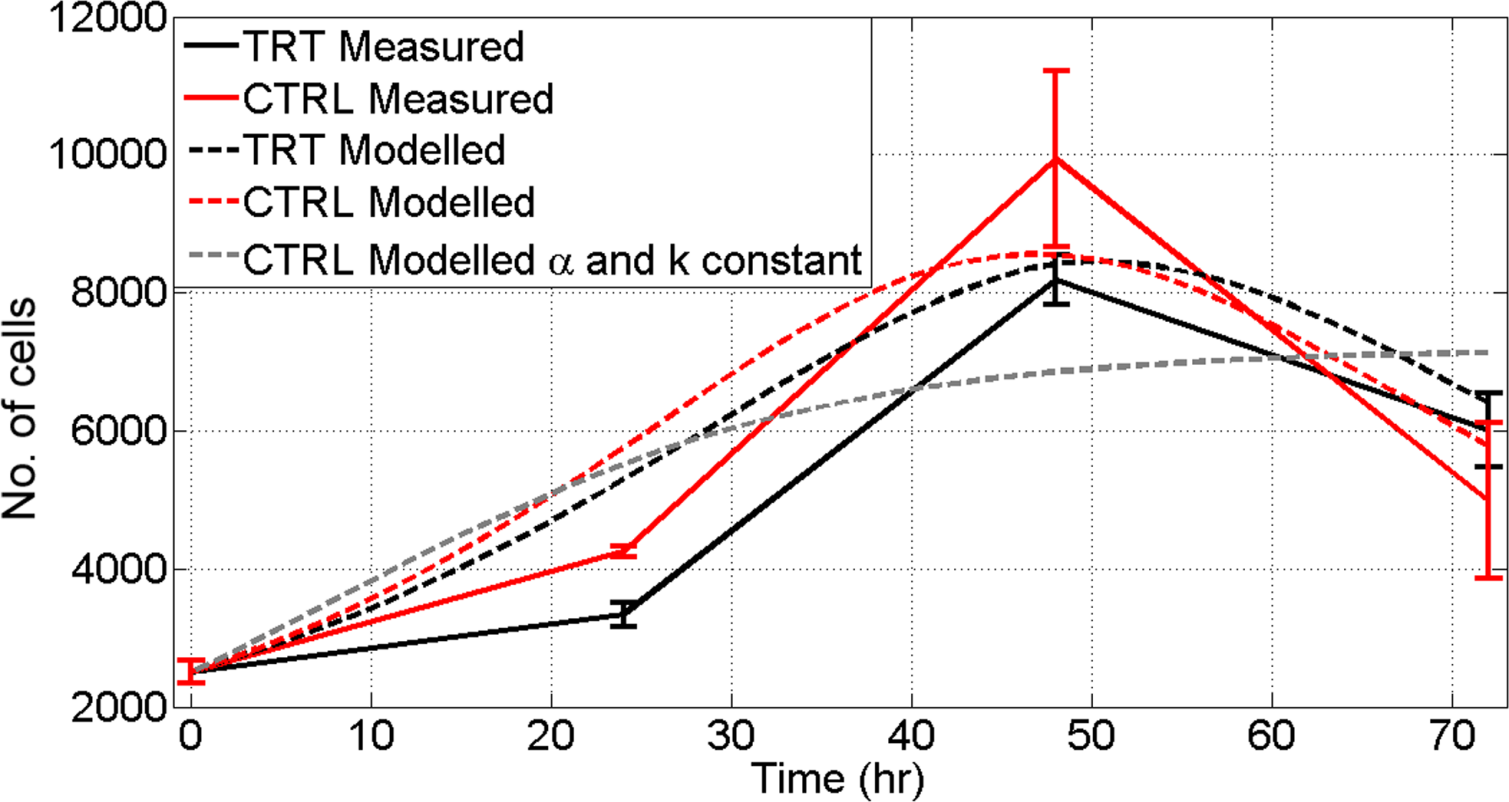
Cell growth simulation models for the A549 cells. The model has been numerically solved for the time interval 0–72 hours in order to reproduce the measured curve of cell proliferation in both the conditions (control and treated cells).

**Fig 6:**
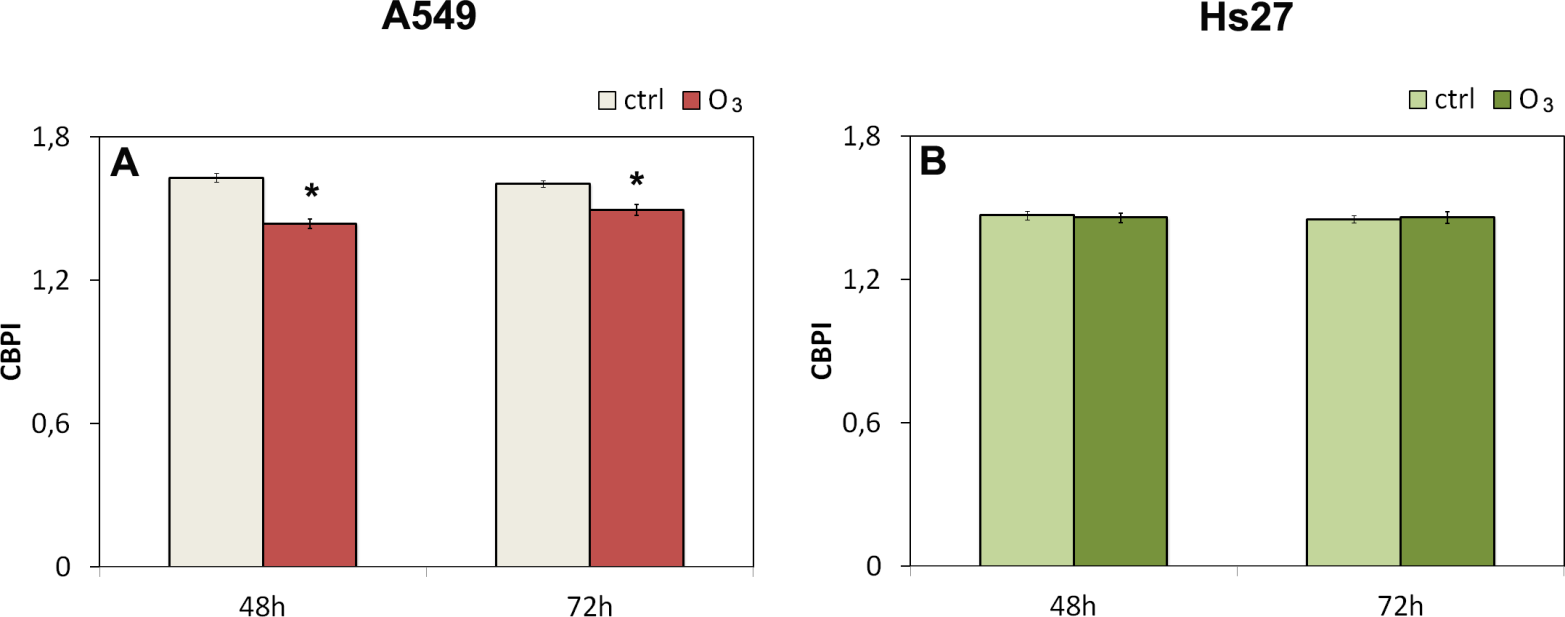
CBPI index in A549 and Hs27 cells 120 ppb O_3_ treated. Cytokinesis Block Proliferation Index (CBPI) in human cells lines A549 and Hs27 treated with 120 ppb O_3_ at 48, 72 hrs. The CBPI indicates the average number of nuclei per cell, and may be used to calculate cell proliferation. CBPI was calculated as follows: (1 × N1) + (2 × N2) + (3 × (N3 + N4))/N where N1–N4 represent the number of cells with 1–4 nuclei, respectively, and N is the total number of cells scored. The results are compared to the negative control and are the means ± ES. * *p* < 0.05.

The model has been numerically solved for the time interval 0–72 hours in order to reproduce the measured curve of cell proliferation in both the conditions (control and treated cells). We found that, using constant values of *α* and *k* the logistic model (dashed grey line in Fig 5) is not able to reproduce the control measured trend (solid red line in Fig 5). In our opinion, this could be due to the fact that our culture system between 48 and 72 hours shows a decrease in the viability differentiating, therefore, from the classic monotonic logistic growth curve. This decrease could be explained considering that, after 48 hours, the growth environment does not supply optimal condition to allow the culture to grow (in terms of space availability, confluence condition). On the contrary, in the second scenario simulation of the A549 control population growth, the model curve (dashed red line in Fig 5) approach significantly better the measurements and it is able to reproduce the measured trend. The parametrization of *α* and *k*, then, improves the model results. To model the genotoxic impact of the O_3_, we utilized the results of the micronuclei assay. The RI index of the A549 cells treated with 120 ppb of O_3_ (Fig 3), in fact, decreases of ~11% after 48 h and of ~6% after 72 h of exposure. We used this information in order to scale the parameters *α*_0_, *β* and *γ*. In details, we assumed that the growth rate *α* is subjected to a decrease (of ~11% for the first 48 h and of ~6% between 48 h and 72 h) accordingly to the decrease of the RI (the slackening in the cells growth is evident also looking at the viability test results in Fig 2 and Fig 6). Moreover, we considered that the carrying capacity *k* decreases exponentially as in the control simulation but with a rate of decrease lower (of ~11% for the first 48 h and of ~6% between 48 h and 72 h). We did this assumption considering that the O_3_ exposure causes a slackening in the replication and, then, the carrying capacity available is greater respect the control scenario. The modelled viability curve of the treated A549 (dashed black line in Fig 5) follows the measured (solid black line in Fig 5) and shows an increase in the doubling time of the cells (the curve is shifted forward respect the control viability trend).

## Discussion

Our experiments were conducted in controlled atmosphere with or without the exposure of 120 ppb of O_3_. The developed method and experimental system is extremely functional in order to evaluate the effects of a single variable (ozone) in repeated exposure experiments as function of the exposition time. Therefore, this system is suitable for a proper quantification, valuation and allocation of the various components involved in the *in vitro* assays under a controlled single variable; the micro environment is characterized by a continuous stream of O_3_ whose concentrations are monitored over time and space. In synthesis, we employed a basilar model of population growth to simulate the cells viability curve for *in vitro* measurements done during the viability assay taking also into account of the atmospheric O_3_ effect in terms of genotoxic damages highlighted by the independent micronuclei test. The concentration of 120 ppb has been chosen since it is observed in several urban areas in Europe and US and represents two times the threshold beyond which the O_3_ short time exposure is a risk for human health and that requires policy intervention (D.Lgs 13 August 2010 in implementation of Directive 2008/50 / EC on ambient air quality).

In previous studies [35] the effects were demonstrated on induction of cell proliferation in the lung after exposure to O_3_ and on mutation fixation and lung tumorigenesis in mice orally exposed to B[*a*]P. Other studies [36,37] showed that lung epithelial cells can become adapted to O_3_ induced cell proliferation upon repeated exposure but cell proliferation indices were higher than those found in untreated control animals. We used in the presented study the human Type-II alveolar adenocarcinoma cell line (A549). This cell line has already been evaluated in several studies and represents a useful model for identification of cytotoxic and mutagenic effects of chemical compounds and emissions [38,39]. For comparison as a normal type cell we used Hs27 normal human dermal fibroblasts. We have demonstrated on the basis of our results *in vitro* that exposure to O_3_ in our experimental model does not alter significantly cellular viability in Hs27 normal human dermal fibroblasts; A549 viability trend in O_3_ treated cells show a significantly decrease at 24 hours vs. control and a decrease at 72 hours vs 48 hours, according to an O_3_ adaptation after the first time exposition as reported in [36,37].

In this work we demonstrated the clastogenic/genotoxic effect of O_3_ on A549 and Hs27 cells. To date studies have been conducted to explore the carcinogenic potential of O_3_ itself [35,40] but no clear evidence exists to link O_3_ exposure to lung cancer development in experimental animals while O_3_ is genotoxic to microorganisms, plants and cell cultures *in vitro* [13]. On the other hand clear evidence of inflammatory and oxidative stress responses to O_3_ is well documented [5–7]. According to our findings the clastogenic/genotoxic effect of ozone on A549 and Hs27 could be related to a possible enhancement of the tumorigenicity of A549. Further studies *in vivo* involving O_3_ exposed human and/or other mammalians could help for the evaluation of risk enhancement and/or development of human alveolar adenocarcinoma of basal epithelial cells. We can keep in mind that yet increased levels of genomic instability related to O_3_ exposure of human lymphocytes *in vivo* and *ex vivo* have been reported [14–16].

In conclusion, we have demonstrated, in our experimental system, that: 1) exposure to 120 ppb of ozone comparable to high pollution days in the urban environment [15] does alter cell viability of A549 but not of Hs27 in respect to the control as showed also by the modelled viability curves 2) 120 ppb O_3_ provokes genotoxic effects more evident in A549 cancer cells than in normal fibroblasts Hs27 ones. Further studies are needed to elucidate the mechanism behind these genotoxic effects in order to facilitate the risks assessment of human alveolar adenocarcinoma. To date lung cancer together with asthma, chronic obstructive pulmonary disease and respiratory infections all seem to be exacerbated because of exposure to ozone, particulate matter and nitrogen oxides [41].

## Supporting information

S1 Table Celltiter viability luminescent. Viability test in A549 and in Hs27 cells.
(PDF)

S2 Table Micronuclei. Micronuclei induction in A549 and Hs27.
(PDF)

S3 Table CBPI and RI. Cytokinesis Block Proliferation Index (CBPI) and Replication Index in A549 and Hs27 cells.
(PDF)

S4 Table Data Comet test. Genotoxic effect of ozone on A549 and Hs27 cells as determined by Comet test.
(XLSX)
