# Supplementary material for: Effects of ozone exposure on human epithelial adenocarcinoma and normal fibroblasts cells
Source: PLoS One. 2017 Sep 8;12(9):e0184519. doi: 10.1371/journal.pone.0184519 (PMC5590931; doi:10.1371/journal.pone.0184519)
Supplement: S1 Table — Viability test in A549 and in Hs27 cells. (PDF) [file pone.0184519.s001.pdf]

CELL PROLIFERATION

**A549**																	
		0,00	MEDIA	DV.ST	ERR.STD	24 h	MEDIA	DV.ST	ERR.STD	48 h	MEDIA	DV.ST	ERR.STD	72 h	MEDIA	DV.ST	ERR.STD
	ctrl 1	8230,00				13790,00				29860,00				14390,00			
		9590,00				15950,00				28970,00				24450,00			
		7660,00	8493,33	991,58	572,49	15610,00	15116,67	1161,44	670,56	52450,00	37093,33	13306,71	7682,63	11980,00	16940,00	6614,54	3818,90
	ctrl2	9212,00				14690,00				29710,00				15790,00			
		7490,00				13942,00				11435,00				30590,00			
		6305,00	7669,00	1461,74	843,94	14510,00	14380,67	390,41	225,40	42750,00	27965,00	15730,26	9081,87	16490,00	20956,67	8350,05	4820,90
	ctrl3	7543,00				14785,00				29520,00				7790,00			
		12500,00				11890,00				26910,00				15990,00			
		7910,00	9317,67	2762,08	1594,69	14890,00	13855,00	1702,55	982,97	52240,00	36223,33	13932,09	8043,70	14990,00	12923,33	4473,63	2582,85
MEAN		8493,33	**8493,33**	824,33	475,93	14450,78	**14450,78**	633,75	365,89	33760,56	**33760,56**	5037,91	2908,64	16940,00	**16940,00**	4016,67	2319,02
DEV.ST		1787,99				1183,74				13179,10				6357,67			
SEM		632,15				418,51				4659,51				2247,77			
			MEDIA	DV.ST	ERR.STD		MEDIA	DV.ST	ERR.STD		MEDIA	DV.ST	ERR.STD		MEDIA	DV.ST	ERR.STD
	trt	8330,00				11710,00				26620,00				22590,00			
		10590,00				12130,00				26940,00				16830,00			
		7670,00	8863,33	1531,32	884,11	10160,00	11333,33	1037,61	599,06	29860,00	27806,67	1785,42	1030,81	21790,00	20403,33	3120,34	1801,53
	trt	8240,00				11840,00				26560,00				24690,00			
		6600,00				15260,00				24990,00				16860,00			
		8840,00	7893,33	1159,54	669,46	10300,00	12466,67	2538,69	1465,71	34940,00	28830,00	5349,33	3088,44	21790,00	21113,33	3958,62	2285,51
	trt	9640,00				12510,00				27590,00				22470,00			
		7790,00				10940,00				21990,00				14910,00			
		8960,00	8796,67	935,75	540,26	7150,00	10200,00	2755,56	1590,92	30780,00	26786,67	4449,72	2569,05	21710,00	19696,67	4162,76	2403,37
MEAN		8517,78	**8517,78**	541,81	312,81	11333,33	**11333,33**	1133,33	654,33	27807,78	**27807,78**	1021,67	589,86	20404,44	**20404,44**	708,33	408,96
DEV.ST		1166,81				2177,61				3699,14				3325,69			
SEM		412,53				769,90				1307,84				1175,81			
**Triton-x-100**																	
			MEAN	DS	SEM		MEAN	DS	SEM		MEAN	DS	SEM		MEAN	DS	SEM
	Contro	9000,00				6725,00				5034,00				4753,00			
		9920,00				5850,00				5120,00				4820,00			
		9120,00	9346,67	500,13	288,75	6820,00	6465,00	534,72	308,72	4980,00	5044,67	70,61	40,76	4070,00	4547,67	415,03	239,62
	Contro	9080,00				7730,00				4100,00				4210,00			
		9120,00				6230,00				5870,00				5020,00			
		9100,00	9100,00	20,00	11,55	8120,00	7360,00	997,85	576,11	5020,00	4996,67	885,23	511,09	5030,00	4753,33	470,57	271,68
	Contro	9090,00				7131,00				5130,00				5030,00			
		8920,00				6200,00				5620,00				5120,00			
		7830,00	8613,33	683,69	394,73	7010,00	6780,33	506,21	292,26	5020,00	5256,67	319,43	184,42	4820,00	4990,00	153,95	88,88
MEAN		9020,00	**9020,00**	373,15	215,44	6868,44	**6868,44**	453,96	262,09	5099,33	**5099,33**	138,35	79,88	4763,67	**4763,67**	221,35	127,80
DEV.ST		532,85				734,19				486,85				375,62			
SEM		188,39				259,57				172,13				132,80			

CELL PROLIFERATION

	**HF**																				
			0,00	MEAN	DS	SEM		24h	MEAN	DS	SEM		48h	MEAN	DS	SEM		72h	MEAN	DS	SEM
		ctrl	17660,00					20080,00					33110,00					24590,00			
			14380,00					19830,00					26760,00					32310,00			
			15600,00	15880,00	1657,83	957,15		25170,00	21693,33	3013,48	1739,83		30040,00	29970,00	3175,58	1833,42		28450,00	28450,00	3860,00	2228,57
		ctrl	17640,00					22100,00					33130,00					24630,00			
			12360,00					19820,00					15780,00					44330,00			
			12700,00	14233,33	2955,15	1706,16		27360,00	23093,33	3866,90	2232,56		30060,00	26323,33	9258,92	5345,64		28470,00	32476,67	#######	6029,44
		ctrl	17490,00					21100,00					34080,00					20520,00			
			17460,00					13710,00					25740,00					34350,00			
			17630,00	17526,67	90,74	52,39		25070,00	19960,00	5765,16	3328,52		41030,00	33616,67	7655,52	4419,92		18400,00	24423,33	8661,85	5000,92
	MEAN		15880,00	**15880,00**	1646,67	950,70		21582,22	**21582,22**	1569,62	906,22		29970,00	**29970,00**	3646,67	2105,40		28450,00	**28450,00**	4026,67	2324,80
	DEV.ST		2214,95					3790,70					6571,21					7418,17			
	SEM		783,10					1340,21					2323,27					2622,72			
				MEAN	DS	SEM			MEAN	DS	SEM			MEAN	DS	SEM			MEAN	DS	SEM
		trt	17640,00					24670,00					26570,00					25810,00			
			14400,00					23890,00					24110,00					25480,00			
			15610,00	15883,33	1637,20	945,24		17490,00	22016,67	3939,56	2274,51		28470,00	26383,33	2185,99	1262,08		28960,00	26750,00	1921,02	1109,10
		trt	17600,00					28690,00					24590,00					29850,00			
			12410,00					33900,00					24130,00					25470,00			
			14520,00	14843,33	2610,06	1506,92		17470,00	26686,67	8396,20	4847,55		28490,00	25736,67	2395,52	1383,06		27850,00	27723,33	2192,75	1265,98
		trt	19690,00					24680,00					26540,00					19870,00			
			14440,00					13890,00					25050,00					23470,00			
			16610,00	16913,33	2638,11	1523,11		13470,00	17346,67	6354,32	3668,67		29500,00	27030,00	2265,10	1307,76		30990,00	24776,67	5673,99	3275,88
	MEAN		15880,00	**15880,00**	1035,00	597,56		22016,67	**22016,67**	4670,00	2696,23		26383,33	**26977,78**	2283,78	1318,54		26416,67	**26416,67**	1501,35	866,80
	DEV.ST		2217,33					6924,96					2055,62					3444,37			
	SEM		783,94					2448,34					726,77					1217,77			
**Triton-x-100**																					
				MEAN	DS	SEM			MEAN	DS	SEM			MEAN	DS	SEM			MEAN	DS	SEM
		Contro	14700,00					8834,00					7034,00					7032,00			
			13690,00					9230,00					7820,00					7132,00			
			12800,00	13730,00	950,63	548,85		8923,00	8995,67	207,76	119,95		8021,00	7625,00	521,59	301,14		6782,00	6982,00	180,28	104,08
		Contro	12900,00					7720,00					6978,00					6987,00			
			16730,00					8129,00					7452,00					8013,00			
			12680,00	14103,33	2277,42	1314,87		9132,00	8327,00	726,53	419,46		6590,00	7006,67	431,71	249,25		6598,00	7199,33	731,01	422,05
		Contro	13780,00					9634,00					6453,00					7024,00			
			13670,00					9703,00					6342,00					7110,00			
			12370,00	13273,33	784,24	452,78		8341,00	9226,00	767,21	442,95		7034,00	6609,67	371,65	214,57		6734,00	6956,00	197,01	113,74
	MEAN		13702,22	**13702,22**	415,70	240,00		8849,56	**8849,56**	466,97	269,61		7080,44	**7080,44**	511,67	295,41		7045,78	**7045,78**	133,62	77,14
	DEV.ST		1343,85					673,39					587,79					405,97			
	SEM		475,12					238,08					207,81					143,53			
