# Supplementary material for: Effects of ozone exposure on human epithelial adenocarcinoma and normal fibroblasts cells
Source: PLoS One. 2017 Sep 8;12(9):e0184519. doi: 10.1371/journal.pone.0184519 (PMC5590931; doi:10.1371/journal.pone.0184519)
Supplement: S2 Table — Micronuclei induction in A549 and Hs27. (PDF) [file pone.0184519.s002.pdf]

TEST MICRONUCLEI

A549 CONTROL		48 h		72 h	
		CELLS BIN.	MICRONUCLEI	CELLS BIN.	MICRONUCLEI
Sample 1	Glass 1	1335	5	1789	6
	Glass 2	1155	10	468	3
	Glass 3	335	2	817	4
Sample 2	Glass 1	1120	7	1650	6
	Glass 2	980	6	923	7
	Glass 3	1100	6	824	6
Sample 3	Glass 1	1400	6	632	4
	Glass 2	1222	7	972	6
	Glass 3	1210	6	1110	9
**1000*micronuclei/binucleated**					
			3,75		3,35
			8,66		6,41
			5,97		4,90
			6,25		3,64
			6,12		7,58
			5,45		7,28
			4,29		6,33
			5,73		6,17
			4,96		8,11
		MEAN	**5,69**	MEAN	**5,97**
		DEV ST	1,40	DEV ST	1,68
		SEM	0,50	SEM	0,60

A549 TRT		48 h		72 h	
		CELLS BIN.	MICRONUCLEI	CELLS BIN.	MICRONUCLEI
Sample 1	Glass 1	68	1	3066	24
	Glass 2	872	14	2666	19
	Glass 3	1371	14	2544	15
Sample 2	Glass 1	1100	12	3112	22
	Glass 2	1012	16	2734	21
	Glass 3	983	13	2671	19
Sample 3	Glass 1	701	11	2523	22
	Glass 2	673	9	1892	19
	Glass 3	989	12	2459	20
**1000*micronuclei/binucleated**					
			14,71		7,83
			16,06		7,13
			10,21		5,90
			10,91		7,07
			15,81		7,68
			13,22		7,11
			15,69		8,72
			13,37		10,04
			12,13		8,13
		MEAN	**13,57**		**7,73**
		DEV ST	2,17		1,17
		SEM	0,77		0,42

Hs27 CONTROL		48 h		72 h	
		CELLS BIN.	MICRONUCLEI	CELLS BIN.	MICRONUCLEI
Sample 1	Glass 1	988	6	887	3
	Glass 2	822	4	564	3
	Glass 3	432	3	656	2
Sample 2	Glass 1	871	4	732	2
	Glass 2	789	6	762	3
	Glass 3	867	5	689	3
Sample 3	Glass 1	982	8	962	2
	Glass 2	1020	4	752	5
	Glass 3	992	5	613	2
**1000*micronuclei/binucleated**					
		**48 h**	**72 h**		
		6,073	3,382		
		4,866	5,319		
		6,944	3,049		
		4,592	2,732		
		7,605	3,937		
		5,767	4,354		
		8,147	2,079		
		3,922	6,649		
		5,040	3,263		
	MEAN	**5,884**	**3,863**		
	DS	1,044	1,226		
	SEM	0,603	0,708		

Hs27 TRT		48 h		72 h	
		BINUCLEATE	MICRONUCLEI	BINUCLEATE	MICRONUCLEI
Sample 1	Glass 1	381	5	428	4
	Glass 2	746	4	860	5
	Glass 3	236	2	490	4
Sample 2	Glass 1	546	3	579	5
	Glass 2	432	7	563	4
	Glass 3	671	5	742	5
Sample 3	Glass 1	602	3	654	6
	Glass 2	534	6	732	4
	Glass 3	523	5	743	5
**1000*micronuclei/binucleated**					
		**48 h**	**72 h**		
		13,123	9,346		
		5,362	5,814		
		8,475	8,163		
		5,495	8,636		
		16,204	7,105		
		7,452	6,739		
		4,983	9,174		
		11,236	5,464		
		9,560	6,729		
	MEAN	**9,099**	**7,463**		
	DS	3,848	1,425		
	SEM	2,222	0,823		
