# Supplementary material for: Effects of ozone exposure on human epithelial adenocarcinoma and normal fibroblasts cells
Source: PLoS One. 2017 Sep 8;12(9):e0184519. doi: 10.1371/journal.pone.0184519 (PMC5590931; doi:10.1371/journal.pone.0184519)
Supplement: S3 Table — Cytokinesis Block Proliferation Index (CBPI) and Replication Index in A549 and Hs27 cells. (PDF) [file pone.0184519.s003.pdf]

CBPI

A549 48 h															
	MONO	BN	Poly	Tot	CBPI		CBPI			A549 48 h Replication Index (RI)				MEAN	SEM
ctrl	338	659	3	1000	1,665	A549 48 h		SEM	ctrl	0,665	1,0000	1,0589	1,0902	**100**	**5,1**
	380	612	8	1000	1,628	ctrl	1,628	0,023		0,628	1,0000	1,0295	0,9444		
	396	598	6	1000	1,61	120 ppm O3	1,557	0,007		0,610	1,0000	0,9173	0,9713		
O3	466	520	14	1000	1,548				O3	0,548	0,8241	0,8726	0,8984	**88,0**	**3,3**
	450	536	14	1000	1,564					0,564	0,8481	0,8981	0,9246		
	451	538	11	1000	1,56					0,56	0,8421	0,8917	0,9180		
A549 72 h										A549 72 h Replication Index (RI)				MEAN	SEM
	MONO	BN	Poly	Tot	CBPI	A549 72 h			ctrl	0,627	1,0000	1,0279	1,0591	**100**	**3,3**
ctrl	374	625	1	1000	1,627	ctrl	1,610	0,014		0,61	1,0000	1,0304	0,9729		
	396	598	6	1000	1,61	120 ppm O3	1,567	0,016		0,592	1,0000	0,9442	0,9705		
	416	576	8	1000	1,592				O3	0,546	0,8708	0,8951	0,9223	**93,1**	**3,5**
O3	479	496	25	1000	1,546					0,57	0,9091	0,9344	0,9628		
	442	546	12	1000	1,57					0,586	0,9346	0,9607	0,9899		
	430	554	16	1000	1,586										
Hs27 48 h															
	MONO	BN	Poly	Tot	CBPI	Hs27 48 h				Hs27 48 h Replication Index (RI)				MEAN	SEM
ctrl	567	423	10	1000	1,443	ctrl	1,490	0,033	ctrl	0,443	1,0000	0,8703	0,8569	**100**	**9,9**
	506	479	15	1000	1,509	120 ppm O3	1,481	0,025		0,509	1,0000	0,9845	1,1490		
	501	481	18	1000	1,517					0,517	1,0000	1,1670	1,0157		
O3	567	420	13	1000	1,446				O3	0,446	1,0068	0,8762	0,8627	**98,6**	**8,6**
	514	472	14	1000	1,5					0,5	1,1287	0,9823	0,9671		
	520	464	16	1000	1,496					0,496	1,1196	0,9745	0,9594		
Hs27 72 h															
	MONO	BN	Poly	Tot	CBPI	Hs27 72 h				Hs27 72 h Replication Index (RI)				MEAN	SEM
ctrl	514	480	6	1000	1,492	ctrl	1,460	0,023	ctrl	0,492	1,0000	1,0933	1,1207	**100**	**6,9**
	558	434	8	1000	1,45	120 ppm O3	1,466	0,049		0,45	1,0000	1,0251	0,9146		
	566	429	5	1000	1,439					0,439	1,0000	0,8923	0,9756		
O3	467	531	2	1000	1,535				O3	0,535	1,0874	1,1889	1,2187	**101,5**	**11,8**
	580	416	4	1000	1,424					0,424	0,8618	0,9422	0,9658		
	569	422	9	1000	1,44					0,44	0,8943	0,9778	1,0023		

A549				
RI 48h	Ctrl	Ozone	SEM ctrl	SEM ozone
1	100	88	5,1	3,3
2	100	88	3,1	4,4
3	100	89,5	4,4	4,3
MEAN	100	88,5	4,2	4
RI 72h				
1	100	93,1	3,3	3,5
2	100	92,3	3	2,7
3	100	91,8	5,6	3,7
MEAN	100	92,4	3,96666667	3,3
Hs27				
RI 48h	Ctrl	Ozone	SEM ctrl	SEM ozone
1	100	98,6	9,9	8,6
2	100	97,2	8,8	7,1
3	100	94,9	7,7	6,4
MEAN	100	96,9	8,8	7,36666667
RI 72h				
1	100	101,5	6,9	11,8
2	100	96,3	8,3	6,1
3	100	95,4	4	5,3
MEAN	100	97,7333333	6,4	7,73333333

A549 48 h										A549 48 h Replication Index (RI)				MEAN	SEM
	MONO	BN	Poly	Tot	CBPI		CBPI		ctrl	0,644	1,0000	1,0047	1,0506	**100**	**3,1**
ctrl	360	636	4	1000	1,644	A549 48 h		SEM		0,641	1,0000	1,0457	0,9953		
	369	621	10	1000	1,641	ctrl	1,641	0,014		0,613	1,0000	0,9519	0,9563		
	398	591	11	1000	1,613	120 ppm O3	1,556	0,025	O3	0,53	0,8230	0,8268	0,8646	**88,0**	**4,4**
O3	485	500	15	1000	1,53					0,59	0,9161	0,9204	0,9625		
	434	542	24	1000	1,59					0,549	0,8525	0,8565	0,8956		
	457	537	6	1000	1,549										
A549 72 h										A549 72 h Replication Index (RI)				MEAN	SEM
	MONO	BN	Poly	Tot	CBPI	A549 72 h			ctrl	0,623	1,0000	1,0349	1,0524	**100**	**3,0**
ctrl	378	621	1	1000	1,623	ctrl	1,606	0,013		0,602	1,0000	1,0169	0,9663		
	400	598	2	1000	1,602	120 ppm O3	1,559	0,011		0,592	1,0000	0,9502	0,9834		
	416	576	8	1000	1,592				O3	0,55	0,8828	0,9136	0,9291	**92,3**	**2,7**
O3	460	530	10	1000	1,55					0,574	0,9213	0,9535	0,9696		
	438	550	12	1000	1,574					0,552	0,8860	0,9169	0,9324		
	458	532	10	1000	1,552										
Hs27 48 h															
	MONO	BN	Poly	Tot	CBPI	Hs27 48 h				Hs27 48 h Replication Index (RI)				MEAN	SEM
ctrl	564	422	14	1000	1,45	ctrl	1,492	0,030	ctrl	0,45	1,0000	0,8841	0,8704	**100**	**8,8**
	506	479	15	1000	1,509	120 ppm O3	1,476	0,017		0,509	1,0000	0,9845	1,1311		
	501	481	18	1000	1,517					0,517	1,0000	1,1489	1,0157		
O3	543	437	20	1000	1,477				O3	0,477	1,0600	0,9371	0,9226	**97,2**	**7,1**
	563	419	18	1000	1,455					0,455	1,0111	0,8939	0,8801		
	522	459	19	1000	1,497					0,497	1,1044	0,9764	0,9613		
Hs27 72 h															
	MONO	BN	Poly	Tot	CBPI	Hs27 72 h				Hs27 72 h Replication Index (RI)				MEAN	SEM
ctrl	508	479	13	1000	1,505	ctrl	1,468	0,028	ctrl	0,505	1,0000	1,0978	1,1503	**100**	**8,3**
	557	426	17	1000	1,46	120 ppm O3	1,449	0,012		0,46	1,0000	1,0478	0,9109		
	566	429	5	1000	1,439					0,439	1,0000	0,8693	0,9543		
O3	548	447	5	1000	1,457				O3	0,457	0,9050	0,9935	1,0410	**96,3**	**6,1**
	576	416	8	1000	1,432					0,432	0,8554	0,9391	0,9841		
	556	430	14	1000	1,458					0,458	0,9069	0,9957	1,0433		

A549 48 h										A549 48 h Replication Index (RI)				MEAN	SEM
	MONO	BN	Poly	Tot	CBPI		CBPI		ctrl	0,663	1,0000	1,0694	1,0659	**100**	**4,4**
ctrl	340	657	3	1000	1,663	A549 48 h		SEM		0,62	1,0000	0,9968	0,9351		
	385	610	5	1000	1,62	ctrl	1,620	0,020		0,622	1,0000	0,9382	1,0032		
	389	600	11	1000	1,622	120 ppm O3	1,568	0,021	O3	0,541	0,8160	0,8726	0,8698	**89,5**	**4,3**
O3	473	513	14	1000	1,541					0,57	0,8597	0,9194	0,9164		
	452	526	22	1000	1,57					0,592	0,8929	0,9548	0,9518		
	423	562	15	1000	1,592										
A549 72 h										A549 72 h Replication Index (RI)				MEAN	SEM
	MONO	BN	Poly	Tot	CBPI	A549 72 h			ctrl	0,641	1,0000	1,0457	1,1014	**100**	**5,6**
ctrl	369	621	10	1000	1,641	ctrl	1,612	0,024		0,613	1,0000	1,0533	0,9563		
	392	603	5	1000	1,613	120 ppm O3	1,561	0,003		0,582	1,0000	0,9080	0,9494		
	426	566	8	1000	1,582				O3	0,557	0,8690	0,9086	0,9570	**91,8**	**3,7**
O3	466	511	23	1000	1,557					0,565	0,8814	0,9217	0,9708		
	452	531	17	1000	1,565					0,561	0,8752	0,9152	0,9639		
	448	543	9	1000	1,561										

Hs27 48 h															
	MONO	BN	Poly	Tot	CBPI	Hs27 48 h				Hs27 48 h Replication Index (RI)				MEAN	SEM
ctrl	503	478	19	1000	1,516	ctrl	1,498	0,027	ctrl	0,516	1,0000	1,1217	0,9981	**100**	**7,7**
	559	422	19	1000	1,46	120 ppm O3	1,471	0,018		0,46	1,0000	0,8897	0,8915		
	501	481	18	1000	1,517					0,517	1,0000	1,0019	1,1239		
O3	564	419	17	1000	1,453				O3	0,453	0,8779	0,9848	0,8762	**94,9**	**6,4**
	553	430	17	1000	1,464					0,464	0,8992	1,0087	0,8975		
	528	449	23	1000	1,495					0,495	0,9593	1,0761	0,9574		
Hs27 72 h															
	MONO	BN	Poly	Tot	CBPI	Hs27 72 h				Hs27 48 h Replication Index (RI)				MEAN	SEM
ctrl	518	469	13	1000	1,495	ctrl	1,476	0,013	ctrl	0,495	1,0000	1,0668	1,0532	**100**	**4,0**
	555	426	19	1000	1,464	120 ppm O3	1,454	0,022		0,464	1,0000	0,9872	0,9374		
	549	432	19	1000	1,47					0,470	1,0000	0,9495	1,0129		
O3	584	408	8	1000	1,424				O3	0,424	0,8566	0,9138	0,9021	**95,4**	**5,3**
	553	431	16	1000	1,463					0,463	0,9354	0,9978	0,9851		
	543	439	18	1000	1,475					0,475	0,9596	1,0237	1,0106		

CBPI

**A549**	48h	72h	SEM 48h	SEM 72h	**Hs27**	48h	72h	SEM 48h	SEM 72h
ctrl1	1,628	1,61	0,023	0,014	ctrl1	1,49	1,481	0,033	0,023
ctrl2	1,641	1,606	0,014	0,013	ctrl2	1,492	1,466	0,03	0,028
ctrl3	1,62	1,612	0,02	0,024	ctrl3	1,498	1,454	0,027	0,013
**MEAN**	1,630	1,60933333	0,019	0,017	**MEAN**	1,49333333	1,467	0,03	0,02133333
**OZONE**	48h	72h			**OZONE**	48h	72h		
Trt1	1,559	1,567	0,007	0,016	Trt1	1,481	1,466	0,025	0,049
Trt2	1,556	1,559	0,025	0,011	Trt2	1,476	1,449	0,017	0,012
Trt3	1,568	1,561	0,021	0,003	Trt3	1,471	1,454	0,018	0,022
**MEAN**	1,561	1,56233333	0,01766667	0,01	**MEAN**	1,476	1,45633333	0,02	0,02766667
